# Zika Virus Transmission — Region of the Americas, May 15, 2015–December 15, 2016

**DOI:** 10.15585/mmwr.mm6612a4

**Published:** 2017-03-31

**Authors:** Juniorcaius Ikejezie, Craig N. Shapiro, Jisoo Kim, Monica Chiu, Maria Almiron, Ciro Ugarte, Marcos A. Espinal, Sylvain Aldighieri

**Affiliations:** ^1^Pan American Health Organization, Washington, DC; ^2^Division of Emergency Operations, Office of Public Health Preparedness and Response, CDC.

Zika virus, a mosquito-borne flavivirus that can cause rash with fever, emerged in the Region of the Americas on Easter Island, Chile, in 2014 and in northeast Brazil in 2015 (*1*). In response, in May 2015, the Pan American Health Organization (PAHO), which serves as the Regional Office of the Americas for the World Health Organization (WHO), issued recommendations to enhance surveillance for Zika virus. Subsequently, Brazilian investigators reported Guillain-Barré syndrome (GBS), which had been previously recognized among some patients with Zika virus disease, and identified an association between Zika virus infection during pregnancy and congenital microcephaly (*2*). On February 1, 2016, WHO declared Zika virus–related microcephaly clusters and other neurologic disorders a Public Health Emergency of International Concern.* In March 2016, PAHO developed case definitions and surveillance guidance for Zika virus disease and associated complications (*3*). Analysis of reports submitted to PAHO by countries in the region or published in national epidemiologic bulletins revealed that Zika virus transmission had extended to 48 countries and territories in the Region of the Americas by late 2016. Reported Zika virus disease cases peaked at different times in different areas during 2016. Because of ongoing transmission and the risk for recurrence of large outbreaks, response efforts, including surveillance for Zika virus disease and its complications, and vector control and other prevention activities, need to be maintained.

## Epidemiologic Surveillance

Data were provided to PAHO by national health authorities under the International Health Regulations or collected from publicly available reports from Ministries of Health. Weekly incidence rates were calculated using 2016 population estimates, except for countries that reported Zika virus circulation in 2015, for which average 2015–2016 population estimates were used.^†^ In this report, case counts for Zika virus and Zika virus–associated GBS represent suspected and laboratory-confirmed cases combined. Depending upon reporting country and territory, epidemiologic week refers either to week of onset or week of report. In Brazil, Zika virus disease became a nationally notifiable condition in February 2016 (*4*); as a result, case counts for 2015 were not available.

**FIGURE 1:**
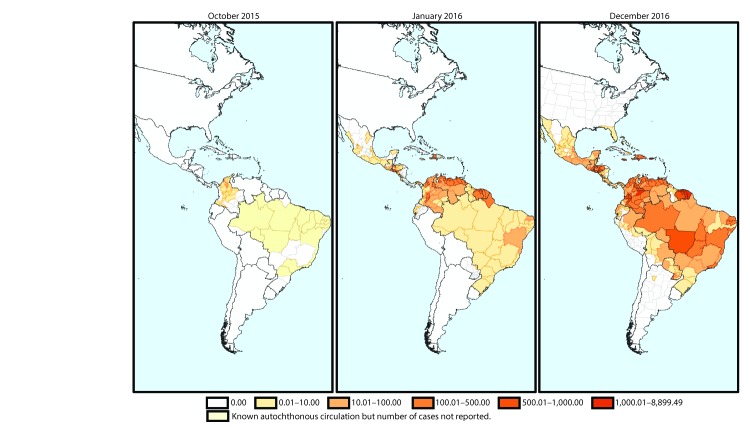
Cumulative suspected and confirmed cases of Zika virus disease per 100,000 population — Region of the Americas,* October 2015, January 2016, and December 2016 * Maps show first-level administrative divisions (states, departments, and provinces) with circulation of Zika virus, as officially reported by national health authorities. Where data on the incidence of Zika virus disease at the subnational level were not available, the national incidence rate was used for the entire country/territory; Zika virus was not necessarily present throughout the entire shaded area.

From May 15, 2015, when Zika virus circulation was confirmed in Brazil, to December 15, 2016, a total of 707,133 autochthonous Zika virus cases were reported in the Region of the Americas, 175,063 (25%) of which were classified as laboratory-confirmed. Autochthonous Zika virus cases had been identified in two countries (Brazil and Colombia) by October 2015 (Figure 1). Zika virus subsequently spread across the Andean subregion,^§^ Central America, and Latin and non-Latin Caribbean. Later in 2016, autochthonous cases were detected in countries in the Southern Cone other than Brazil and parts of North America. As of December 15, 2016, local transmission had been reported in 48 countries and territories^¶^ in the Region of the Americas.

**FIGURE 2:**
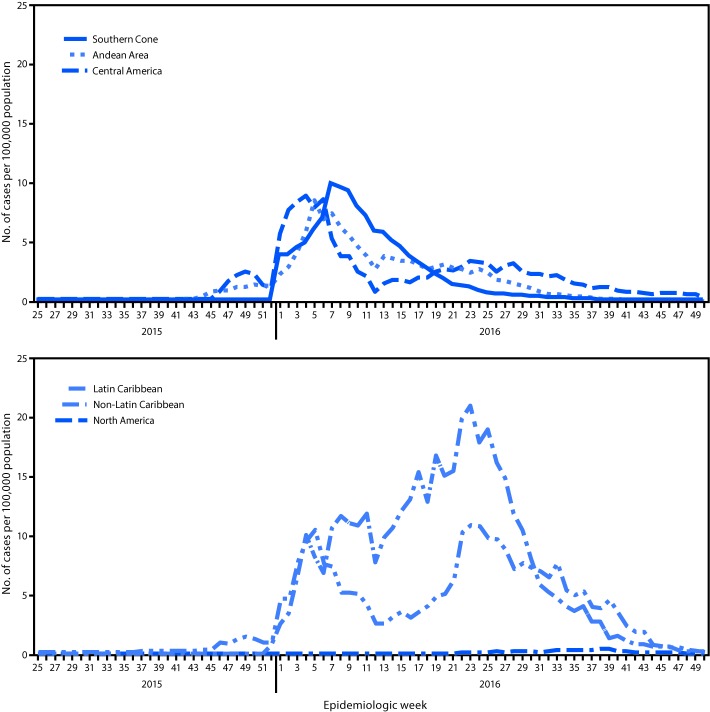
Suspected and confirmed cases of Zika virus disease per 100,000 population, by subregion* and epidemiologic week — Region of the Americas, May 2015–December 2016 * The following countries and territories reporting Zika virus disease cases by epidemiologic week were included in this figure.** Southern Cone:** Brazil; Paraguay. **Andean: **Bolivia; Colombia; Ecuador; Peru; Venezuela. **Central America:** Belize; Costa Rica; El Salvador; Guatemala; Honduras; Panama. **Non-Latin Caribbean:** Anguilla; Antigua and Barbuda; Aruba; Barbados; Bonaire, Sint Eustatius, and Saba; British Virgin Islands; Cayman Islands; Dominica; Grenada; Guyana; Jamaica; Montserrat; Saint Kitts and Nevis; Saint Vincent and the Grenadines; Sint Maarten; Suriname; Trinidad and Tobago; Turks and Caicos. **Latin Caribbean:** Dominican Republic; French Guiana; Guadeloupe; Haiti; Martinique; Saint Barthélemy; Saint Martin. **North America:** Mexico.

From May 15, 2015, to December 15, 2016, rates of Zika virus disease peaked at different times in different subregions of the Americas (Figure 2). In both the Southern Cone and Andean subregions, rates increased in January, peaked in February, and progressively declined. In Central America, rates peaked in January, followed by a more modest peak in June. In the non-Latin Caribbean, incidence peaks of comparable intensity were reported in February and June. In the Latin Caribbean subregion, where the highest rates of reported Zika virus disease cases were observed, rates began to increase in January 2016, and continued at high levels through July. Reported rates remained relatively low in North America.

**FIGURE 3:**
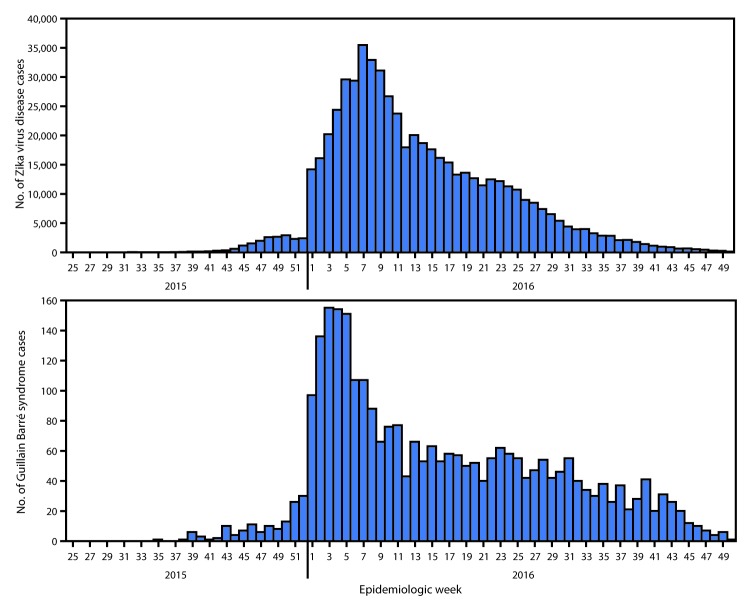
Suspected and confirmed cases of Zika virus* and Guillain-Barré syndrome,^†^ by epidemiologic week — Region of the Americas, May 2015–December 2016 * The following countries and territories reporting Zika virus disease cases by epidemiologic week were included in this figure: Anguilla, Antigua and Barbuda, Aruba, Barbados, Belize, Bolivia, Bonaire, St Eustatius, and Saba, Brazil, Cayman Islands, Colombia, Costa Rica, Dominica, Dominican Republic, Ecuador, El Salvador, French Guiana, Grenada, Guadeloupe, Guatemala, Guyana, Haiti, Honduras, Jamaica, Martinique, Mexico, Montserrat, Panama, Paraguay, Peru, Saint Barthelemy, Saint Kitts and Nevis, Saint Vincent and the Grenadines, Sint Maarten, St. Martin, Suriname, Trinidad and Tobago, Turks and Caicos Islands, Venezuela, British Virgin Islands ^†^ The following countries and territories reporting Guillain-Barré syndrome cases by epidemiologic week were included in this figure: Barbados, Belize, Bolivia, Colombia, Costa Rica, Dominica, Dominican Republic, Ecuador, El Salvador, Grenada, Guadeloupe, Guatemala, Haiti, Honduras, Jamaica, Martinique, Mexico, Panama, Paraguay, Puerto Rico, Saint Vincent and the Grenadines, Suriname, Venezuela.

As of December 15, 2016, increases in the number of GBS cases had been reported in 13 countries and territories with documented Zika virus transmission, compared with baseline data.** Six additional countries and territories reported laboratory confirmation of Zika virus infection in at least one GBS patient. The temporal trend in reported GBS cases in the 19 countries has largely paralleled that of Zika virus disease cases (Figure 3). Although congenital microcephaly and other neurologic abnormalities have been reported among infants born to mothers who were infected with Zika virus during pregnancy (*5*), variable reporting of congenital Zika virus syndrome did not permit a comparison of trends in reported congenital abnormalities within the region.

## Public Health Response

In December 2015, PAHO activated an incident management system to coordinate the regional Zika virus response and developed a framework for action with four pillars: 1) detection of Zika virus and its complications, 2) prevention of new infections, 3) provision of care and support for affected persons and families, and 4) implementation of research to understand the disease and its consequences (*6*). Surveillance and laboratory testing guidelines were issued to assist national authorities in the detection of Zika virus disease cases and associated complications (*3*). In collaboration with CDC, PAHO distributed diagnostic tools, including Trioplex kits for molecular detection and reagents for serologic testing, to 26 countries and territories. Multicountry workshops were organized to provide training in surveillance and laboratory diagnosis.

As of December 15, 2016, in collaboration with the Global Outbreak Alert and Response Network, 86 missions had been conducted in 30 countries and territories during which technical experts, including epidemiologists, entomologists, and virologists, worked with national and local authorities to implement Zika virus control and prevention measures. Assistance was provided to PAHO countries for the implementation of comprehensive health care and social services for infants with congenital abnormalities. PAHO also supported the development of a Zika virus research agenda and standardized protocols to conduct epidemiologic investigations to characterize and evaluate the risk for Zika virus–associated complications (*6**–**7*).

## Discussion

Since the emergence of Zika virus in Brazil, the number of countries and territories reporting Zika virus disease cases has quickly increased in the Region of the Americas. Several factors might have contributed to this rapid spread. The absence of previous reports of Zika virus disease outbreaks in the region suggests that populations were immunologically naïve. The presence of *Aedes aegypti* mosquitoes in most countries and territories of the Region of the Americas facilitated widespread establishment of local transmission. In addition, high levels of travel within the region might have promoted spread to previously unaffected areas.

After reporting high numbers of Zika virus disease cases during the first half of 2016, incidence in all PAHO subregions declined. Reasons for the decline might include the reduction in the number of susceptible persons and seasonal or meteorologic changes, especially in areas with a nontropical climate, leading to lower density of *Ae. aegypti*. Variations in these factors among countries might have resulted in the observed subregional differences in incidence patterns.

In this analysis, the temporal pattern of reported Zika virus disease cases paralleled that of GBS cases, a pattern that has been previously reported (*8*) and which has suggested an association between Zika virus and GBS. The relationship between Zika virus infection during pregnancy and the occurrence of congenital abnormalities has been established (*9*). As knowledge in this area evolves, birth defects surveillance will need to adapt to include newly identified abnormalities associated with Zika virus infection.

Zika virus transmission in the Region of the Americas is ongoing, but as of December 15, 2016, it has decreased in intensity. It is expected that the virus will continue to spread and potentially reach all areas where *Ae. aegypti* mosquitoes are present. The future of Zika virus outbreaks is uncertain; however, recurrent outbreaks caused by other *Aedes*-transmitted arboviruses, including dengue and chikungunya, suggest that Zika virus outbreaks might also continue to occur. Additional research is needed to determine whether transmission in animal populations occurs in the Region of the Americas that might contribute to transmission in humans.

The findings in this report are subject to at least four limitations. First, countries and territories varied in their implementation of PAHO’s case definitions, laboratory testing, and case reporting procedures. A majority reported all detected cases, whereas a few reported only laboratory-confirmed cases, and several countries and territories reported cases before PAHO’s development of standardized case definitions, which made it difficult to determine the exact incidence of Zika virus disease. Second, given the similarities in clinical presentation, an unknown number of suspected cases could have been caused by other arboviruses, which might have led to an overestimation of cases. Third, certain countries and territories did not provide weekly reports of cases, and some reported cases by date of onset, whereas others reported cases by date of notification; these differences might have affected the overall shape of the epidemic curves. Finally, in some areas, results might have been affected by incomplete or delayed reporting from subnational to national levels related to the differences in time it took for countries to build capacity for Zika virus surveillance and laboratory testing.

On November 18, 2016, WHO declared that Zika virus and associated complications remain a considerable public health challenge requiring long-term coordinated action, but no longer represent a Public Health Emergency of International Concern.^††^ Because of ongoing transmission, occurrence of associated complications, and risk for recurrence of large outbreaks, countries and territories in the Region of the Americas and other regions where competent vectors are present need to continue surveillance for Zika virus disease and its complications and implementation of prevention and control measures.

The public health response to Zika virus, a flavivirus not previously recognized in the Region of the Americas, has been particularly challenging because of limited knowledge about the virus, modes of transmission, and associated complications. Difficulties in implementing effective vector-control measures and the absence of antiviral drugs or vaccines have further complicated response efforts. The establishment of national surveillance systems and laboratory testing and implementation of prevention and control measures have been critical for the response. Limiting Zika virus transmission and preventing its associated complications will require continued implementation of comprehensive arboviral disease surveillance, strengthening of surveillance for birth defects and neurologic complications, and continuation of vector control and other prevention activities.

**Summary**

**What is already known about this topic?**

Zika virus, a flavivirus that is primarily transmitted by *Aedes* mosquitoes, has rapidly spread throughout the Region of the Americas since 2015. Zika virus infection during pregnancy is a known cause of microcephaly and other congenital abnormalities, and infection is also associated with neurologic disorders, including Guillain-Barré syndrome (GBS).

**What is added by this report?**

During May 15, 2015–December 15, 2016, autochthonous Zika virus transmission was confirmed in 48 countries and territories in the Region of the Americas. Rates of Zika virus disease peaked at different times in different subregions. During this period, the trend in reported GBS cases paralleled that of reported Zika virus disease cases.

**What are the implications for public health practice?**

Because of ongoing Zika virus transmission, the occurrence of associated complications, and the risk for recurrence of large outbreaks, countries where *Aedes* mosquitoes are present should continue surveillance for Zika virus disease, GBS, and congenital abnormalities; strengthen capacity for laboratory diagnosis of Zika virus and other arboviruses; and continue the implementation of vector control measures and other prevention activities.

## Footnotes

* http://www.who.int/mediacentre/news/statements/2016/1st-emergency-committee-zika/en/.

† https://esa.un.org/unpd/wpp/index.htm.

§ *Southern Cone:* Argentina; Brazil; Paraguay. *Andean:* Bolivia; Colombia; Ecuador; Peru; Venezuela. *Central America:* Belize; Costa Rica; El Salvador; Guatemala; Honduras; Nicaragua; Panama. *Non-Latin Caribbean:* Anguilla; Antigua and Barbuda; Aruba; Bahamas; Barbados; Bonaire, Sint Eustatius, and Saba; British Virgin Islands; Cayman Islands; Curaçao; Dominica; Grenada; Guyana; Jamaica; Montserrat; Saint Kitts and Nevis; Saint Lucia; Saint Vincent and the Grenadines; Sint Maarten; Suriname; Trinidad and Tobago; Turks and Caicos; U.S. Virgin Islands. *Latin Caribbean:* Cuba; Dominican Republic; French Guiana; Guadeloupe; Haiti; Martinique; Puerto Rico; Saint Barthélemy; Saint Martin. *North America:* Mexico; United States.

** Countries and territories that have reported an increase in the incidence of GBS and laboratory confirmation of Zika virus infection in at least one patient with GBS: Brazil; Colombia; Dominican Republic; El Salvador; French Guiana; Guadeloupe; Guatemala; Honduras; Jamaica; Martinique; Puerto Rico; Suriname; Venezuela. Countries and territories that have reported laboratory confirmation of Zika virus infection in at least one patient with GBS: Bolivia; Costa Rica; Grenada; Haiti; Mexico; Panama.

†† http://www.who.int/mediacentre/news/statements/2016/zika-fifth-ec/en/.
